# Update der ESC-Vorhofflimmerleitlinien 2024: Aus der Kommission für Klinische Kardiovaskuläre Medizin der DGK

**DOI:** 10.1007/s00399-025-01096-4

**Published:** 2025-07-31

**Authors:** Renate B. Schnabel, Melanie Anuscha Gunawardene, Christian Andreas Perings, Daniel Steven, Hans-Jörg Busch, Isabel Deisenhofer, Karl Georg Häusler, Philipp Sommer, Ralf Birkemeyer, Lars Eckardt

**Affiliations:** 1 Allgemeine und Interventionelle Kardiologie, Universitäres Herz- und Gefäßzentrum Hamburg, Hamburg, Deutschland; 2 CCB-Cardioangiologisches Centrum Bethanien Frankfurt, Markuskrankenhaus, Frankfurt am Main, Deutschland; 3 Medizinische Klinik I, KLW St. Paulus GmbH, Lünen, Deutschland; 4 Elektrophysiologie, Herzzentrum der Universität zu Köln, Köln, Deutschland; 5 Zentrum für Notfall- und Rettungsmedizin, Universitätsklinikum Freiburg, Freiburg, Deutschland; 6 Abteilung für Elektrophysiologie, TUM Universitätsklinikum Deutsches Herzzentrum, Technische Universität München, München, Deutschland; 7 Klinik für Neurologie, Universitätsklinikum Ulm, Ulm, Deutschland; 8 Klinik für Elektrophysiologie/Rhythmologie, Herz- und Diabeteszentrum NRW, Bad Oeynhausen, Deutschland; 9 Cardiologicum Herzklinik Ulm MVZ, Ulm, Deutschland; 10 Klinik für Kardiologie II – Rhythmologie, Universitätsklinikum Münster, Münster, Deutschland; 11 Kommission für Klinische Kardiovaskuläre Medizin, Deutsche Gesellschaft für Kardiologie, Düsseldorf, Deutschland; 12 Universitäres Herz- und Gefäßzentrum, Klinik für Kardiologie, Universitätsklinikum Hamburg-Eppendorf, Martinistraße 52, Gebäude Ost 50, 20246 Hamburg, Deutschland

**Keywords:** Vorhofflimmern, Behandlung, Leitlinien, Zusammenfassung, Atrial fibrillation, Management, Guidelines, Summary

## Abstract

Turnusmäßig sind die aktualisierten Leitlinien der European Society of Cardiology (ESC) zum Management von Vorhofflimmern 2024 erschienen. Trotz bedeutender Fortschritte in der Prävention, Diagnostik und Therapie ist das Vorhofflimmern die häufigste anhaltende Rhythmusstörung und hat einen großen Einfluss auf die Betroffenen, ihr Umfeld und das Gesundheitswesen. Die prägnanten Leitlinien fokussieren auf evidenzgestützte Empfehlungen und praktische Umsetzbarkeit einschließlich klarer Schaubilder und Behandlungspfade. Die ganzheitliche Betreuung des Patienten und seiner Komorbiditäten wird in einem umfassenden AF-CARE-Konzept in den Vordergrund gestellt. Es beinhaltet „C“ Komorbiditäten und Risikofaktorenmanagement, „A“ Vermeidung von Schlaganfall und Thrombembolien, „R“ Reduktion von Symptomen durch Rhythmus- oder Frequenzkontrolle und dynamische „E“ Re-Evaluation. Bei paroxysmalem Vorhofflimmern ist die Ablation jetzt Erstlinientherapie. Aktuelle Daten für Device-detektiertes Vorhofflimmern und Trigger-induziertes Vorhofflimmern wurden aufgenommen. Eine maximale Involvierung von Patienten u. a. durch parallel publizierte Patientenleitlinien wird zur gemeinsamen, informierten Entscheidungsfindung befürwortet.

## Abstract (en)

In 2024, the current ESC guidelines for the management of atrial fibrillation (AF) were published. Despite significant advances in the prevention, diagnosis and treatment of AF, it remains the most common sustained arrhythmia with a significant impact on patients, their caregivers and public health. The concise guidelines focus on evidence-based recommendations and their practical implementation supported by clear figures and treatment pathways. The holistic care for AF patients including their comorbidities is prioritized in a comprehensive AF-CARE concept. It comprises “C” for comorbidities and risk factor management, “A” for avoidance of stroke and thromboembolism, “R” for reduction of symptoms by rate and rhythm control and “E”, evaluation and dynamic reassessment. Ablation is now the first-line therapy in paroxysmal AF. Current data on device detected AF and triggered AF are considered. Maximum involvement of the patient for informed, shared decision-making is suggested, supported by the parallel publication of patient guidelines.

Die 2024er ESC-Leitlinien zum Vorhofflimmern stellen die ganzheitliche Betreuung des Patienten in den Vordergrund. Das AF-CARE-Konzept beinhaltet neben der Adressierung von Komorbiditäten, Vermeidung von Schlaganfällen/Thromboembolien und Symptomreduktion eine dynamische Reevaluation. Die Rhythmuskontrolle wird bei paroxysmalem Vorhofflimmern und individuell bei symptomatischem Vorhofflimmern priorisiert. Zur Prävention von Schlaganfällen sollen direkte orale Antikoagulanzien Vitamin-K-Antagonisten vorgezogen werden.

Trotz der Fortschritte in der Prävention, Diagnostik und Therapie ist Vorhofflimmern nach wie vor die häufigste therapiebedürftige Arrhythmie in der Bevölkerung mit Einfluss auf die Betroffenen, ihr Umfeld und das Gesundheitssystem [5, 17, 30]. Die 2024er Leitlinien der Europäischen Gesellschaft für Kardiologie (ESC) umfassen 130 praxisorientierte Empfehlungen sowie eine parallel veröffentlichte Patientenversion (https://www.escardio.org/static-file/Escardio/Guidelines/Documents/ESC-Patient-Guidelines-Atrial-Fibrillation.pdf) [29]. Der folgende Artikel gibt eine Übersicht über die wesentlichen Neuerungen in den Leitlinien (Abb. 1; [22]).

**Abb. 1:**
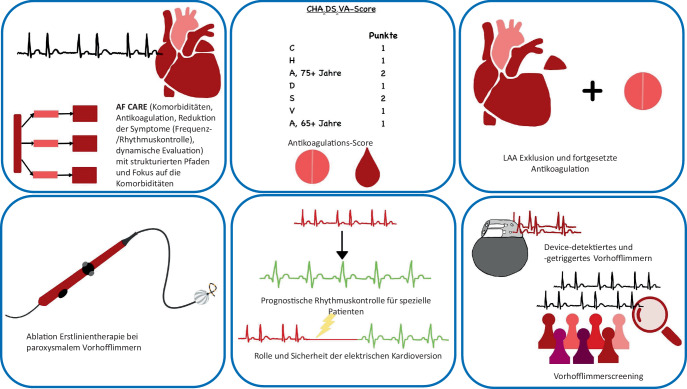
Übersicht über die wichtigsten Neuerungen der 2024er Vorhofflimmerleitlinien der European Society of Cardiology[24]

## Grundzüge des AF-CARE-Konzepts

**Abb. 2:**
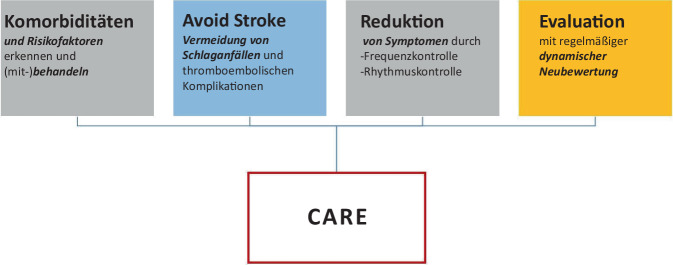
Das AF-CARE-Konzept (siehe auch [24])

Der AF-CARE-Ansatz erweitert frühere Therapiekonzepte zum Vorhofflimmern und gliedert das Management in vier zentrale Säulen, die die evidenzbasierte Behandlung von Vorhofflimmern im Kontext der individuellen Bedürfnisse der Patienten sieht (Abb. 2). Schlüsselelemente sind:

- C, comorbidities: an erster Stelle die Behandlung von Begleiterkrankungen und Risikofaktoren
- A, avoid: Vermeidung von Schlaganfall und Thromboembolien i. d. R. durch Antikoagulation
- R, reduce: Symptomreduktion durch Rhythmus- und Frequenzkontrolle
- E, evaluation: Evaluierung und dynamische Neubeurteilung.

Das systematische und auf den Patienten ausgerichtete AF-CARE-Konzept berücksichtigt eine individualisierte, auf die Bedürfnisse des Patienten zugeschnittene Vorgehensweise bei der Behandlung der Arrhythmie [13]. Zudem wird der dynamischen Natur der Erkrankung, den Komorbiditäten und Risikofaktoren sowie der ständigen Verbesserung der Behandlungsoptionen von Vorhofflimmern Rechnung getragen für eine optimale Therapie.

## Erkennung und Behandlung von Begleiterkrankungen und Risikofaktoren

Die Leitlinien enthalten aktualisierte, strukturierte Behandlungspfade für Patienten mit Vorhofflimmern, die auf die zeitlichen Muster des Vorhofflimmerns – erstmalig diagnostiziert, paroxysmal, persistierend und permanent – zugeschnitten sind. Eine interaktive App für Mobilgeräte ermöglicht einen einfachen und praktischen Zugriff auf die Leitlinien und wird kostenlos zur Verfügung gestellt (https://www.escardio.org/Guidelines/Clinical-Practice-Guidelines/Guidelines-derivativeproducts/ESC-Mobile-Pocket-Guidelines).

Ein wesentlicher Aspekt der Leitlinien 2024 ist die Betonung der Behandlung von Begleiterkrankungen und Risikofaktoren. Für alle Begleiterkrankungen mit ausreichender Evidenzlage, wie Hypertonie, Herzinsuffizienz, Diabetes mellitus, Adipositas, obstruktives Schlafapnoesyndrom, körperliche Aktivität und Alkoholkonsum, werden Ziele für die Behandlung der mit Vorhofflimmern assoziierten Erkrankungen und Risikofaktoren festgelegt. Weitere (kardiale und nichtkardiale) Begleiterkrankungen, die mit Vorhofflimmern in Zusammenhang stehen und die Behandlung beeinflussen können, sollten ebenso individuell berücksichtigt werden. Eine effektive Behandlung von Begleiterkrankungen und Risikofaktoren verbessert die Symptome und die Lebensqualität, verringert die Häufigkeit von Vorhofflimmerrezidiven und verlangsamt den Progress von Vorhofflimmern [16, 21].

## Antikoagulation unter Verwendung lokal validierter Risikoscores oder des CHA_2_DS_2_-VA-Scores

Vorhofflimmern erhöht das Risiko für Thromboembolien, einschließlich ischämischer Schlaganfälle, auch bei paroxysmalem Auftreten [17]. Ohne adäquate Behandlung ist das Risiko für einen ischämischen Schlaganfall bei Patienten mit Vorhofflimmern bis zu fünfmal höher [14], und jeder fünfte Schlaganfall ist auf Vorhofflimmern zurückzuführen. Das Vorliegen eines erhöhten Schlaganfallrisikos hängt nicht von der Art des Vorhofflimmerns oder dem aktuellen Rhythmus ab. Auch wenn der Sinusrhythmus erhalten bleibt oder keine Symptome im Zusammenhang mit Vorhofflimmern auftreten, besteht ein erhöhtes Schlaganfallrisiko. Tatsächlich gibt es Hinweise darauf, dass es keinen direkten zeitlichen Zusammenhang zwischen dem Auftreten eines ischämischen Schlaganfalls und Vorhofflimmern gibt [3, 26]. Angesichts dieses erheblichen Risikos wird für alle geeigneten Patienten mit einem erhöhten Risiko für Thromboembolien eine orale Antikoagulation, vorzugsweise ein direktes orales Antikoagulans (DOAK) empfohlen [23]. Die Wirksamkeit der oralen Antikoagulation zur Prävention von ischämischen Schlaganfällen bei Patienten mit Vorhofflimmern ist gut belegt. Eine alleinige Thrombozytenaggregationshemmung ist für die Schlaganfallprävention bei Patienten mit Vorhofflimmern unverändert zu den vorherigen Leitlinien nicht indiziert.

Eine wichtige Änderung bei der Einschätzung des Schlaganfallrisikos und der Einleitung einer oralen Antikoagulation ist die Nutzung validierter lokaler Risikoscores oder der Einsatz des CHA_2_DS_2_-VA-Scores anstelle des CHA_2_DS_2_-VASc-Scores. Für die bekannten Schlaganfallrisikofaktoren werden folgende Punkte vergeben: Herzinsuffizienz (1 Punkt), Hypertonie (1 Punkt), Alter ≥ 75 Jahre (2 Punkte), Diabetes mellitus (1 Punkt), Vorhofflimmern, Schlaganfall oder transitorische ischämische Attacke (2 Punkte), vaskuläre Erkrankung (1 Punkt) und Alter zwischen 65 und 74 Jahren (1 Punkt). Bei der CHA_2_DS_2_-VA-Score-Berechnung wird das Geschlecht nicht mehr berücksichtigt. Das Geschlecht ist bei Patienten für die Entscheidung zur Antikoagulation nicht mehr ausschlaggebend [4]. Zur Vereinfachung wurden im Vergleich zu den bisherigen Leitlinien die geschlechtsabhängigen Empfehlungen herausgenommen. Mit der Änderung des CHA_2_DS_2_-VA-Scores wird die Beurteilung des Schlaganfallrisikos vereinfacht. Unabhängig vom Geschlecht gelten einheitliche Schwellenwerte: Bei einem CHA_2_DS_2_-VA-Score ≥ 2 wird eine orale Antikoagulation mit einem DOAK empfohlen, sofern der Patient keine Herzklappenprothese oder eine mittel-hochgradige Mitralklappenstenose hat. Bei einem CHA_2_DS_2_-VA-Score von 1 sollte eine orale Antikoagulation erwogen werden. Eine regelmäßige Reevaluation des thromboembolischen Risikos wird im Krankheitsverlauf empfohlen.

Eine Antikoagulation erhöht das Blutungsrisiko. Von der Nutzung von Blutungsrisikoscores zur Entscheidungsfindung bezüglich der Verschreibung von Gerinnungshemmern wird abgeraten. Stattdessen sollen modifizierbare Risikofaktoren für Blutungen regelmäßig überprüft und adressiert werden.

## Ischämischer Schlaganfall unter Antikoagulation

Es ist bekannt, dass eine orale Antikoagulation das Risiko für einen ischämischen Schlaganfall bei Patienten mit Vorhofflimmern deutlich senkt, jedoch verbleibt ein Restrisiko [25]. Dies kann mit Faktoren wie anderen Schlaganfallmechanismen, mangelnder Therapieadhärenz, unzureichender Dosierung oder unzureichender Antikoagulation zusammenhängen.

Bei Patienten mit ischämischem Schlaganfall unter oraler Antikoagulation aufgrund von Vorhofflimmern wird eine umfassende Diagnostik empfohlen. Diese sollte eine Untersuchung nicht kardioembolischer Ursachen, der Risikofaktoren für vaskuläre Erkrankungen, der Dosierung der Medikation und der diesbezüglichen Compliance umfassen, um weitere vaskuläre Ereignisse zu verhindern [11]. Zusätzlich wird in den Leitlinien davon abgeraten, eine Plättchenhemmung zur oralen Antikoagulation hinzuzufügen, um einen erneuten Schlaganfall zu verhindern, da dies mit einem erhöhten Blutungsrisiko einhergeht und keinen nachgewiesenen Nutzen hat (Klasse-III-Empfehlung; [18]). Der Wechsel von einem Vitamin-K-Antagonisten auf ein DOAK kann für bestimmte Patientengruppen sinnvoll sein. Von einem routinemäßigen Wechsel von einem DOAK zu einem anderen oder von einem DOAK zu einem Vitamin-K-Antagonisten ohne klare Indikation wird abgeraten (ebenfalls Klasse-III-Empfehlung; [20]).

Eine Dosisreduktion bei einem DOAC sollte nur bei Erfüllung der medikamentenspezifischen Dosisreduktionskriterien erfolgen, z. B. bei Nierenfunktionseinschränkung. Eine inadäquate Dosisreduktion kann das Schlaganfallrisiko erhöhen, ohne das Blutungsrisiko relevant zu reduzieren [6].

## Vorhofohrverschluss

Das linke Vorhofohr ist seit Langem als Target für die Prävention von Schlaganfällen bei Patienten mit Vorhofflimmern bekannt, da vermutlich ca. 90 % der mit Vorhofflimmern einhergehenden Thromben im linken Vorhofohr ihren Ursprung haben. Die zusätzliche Exklusion des linken Vorhofohrs bei Patienten mit Vorhofflimmern, die sich einer Herzoperation unterziehen, reduziert die Inzidenz von ischämischem Schlaganfall oder Thromboembolien um ein Drittel unter regelhaft fortgesetzter oraler Antikoagulation (LAOOS-III-Studie; [32]). Dabei können verschiedene Techniken (Amputation mit Nahtverschluss, Klammern oder Verschluss mit einem epikardialen Device) zum Einsatz kommen. Aufgrund der vorliegenden Daten soll die Exklusion des linken Vorhofs als Zusatzmaßnahme zur oralen Antikoagulation bei Patienten mit Vorhofflimmern, die sich einer Herzoperation unterziehen, erwogen werden, um ischämische Schlaganfälle und Thromboembolien zu verhindern.

Der Vorhofohrverschluss kann auch während einer endoskopischen oder Hybrid-Katheterablation von Vorhofflimmern mit Hilfe externer Clip-Systeme durchgeführt werden. Beobachtungsstudien haben gezeigt, dass der Vorhofohrverschluss während einer thorakoskopischen Katheterablation mit 95 % vollständigem Verschluss durchführbar und sicher ist und mit einer niedriger als erwarteten Thromboembolierate einhergeht, wenn die Patienten nach der Operation eine orale Antikoagulation erhalten. Unter Berücksichtigung der vorliegenden nichtrandomisierten Daten wird empfohlen, dass bei Patienten mit Vorhofflimmern, die eine endoskopische oder Hybrid-Katheterablation erhalten, der Vorhofohrverschluss als Ergänzung zur oralen Antikoagulation in Betracht gezogen werden sollte zur Verhinderung von thromboembolischen Ereignissen [1]. Bei Patienten mit Kontraindikationen zur langfristigen oralen Antikoagulation kann der perkutane Vorhofohrverschluss zur Verhinderung von ischämischen Schlaganfällen und Thromboembolien ebenso in Betracht gezogen werden. Zum möglichen Nutzen u. a. des interventionellen Vorhofohrverschlusses werden in den kommenden Jahren Ergebnisse randomisierter Studien erwartet.

## Katheterablation

Die katheterbasierte Ablation ist eine etablierte, invasive Behandlungsmethode für Vorhofflimmern. Die Katheterablation ist als Behandlungsmethode der Wahl bei Patienten mit paroxysmalem oder persistierendem Vorhofflimmern, die auf Antiarrhythmika nicht ansprechen oder diese nicht vertragen, etabliert. In den aktuellen Leitlinien wird die Katheterablation aufgrund der Evidenzlage aufgewertet als Erstlinientherapie zur Rhythmuskontrolle bei Patienten mit paroxysmalem Vorhofflimmern, die noch keine Antiarrhythmika erhalten haben. Diese Empfehlung wird durch mehrere randomisierte kontrollierte Studien gestützt, die belegen, dass die Katheterablation im Vergleich zu Antiarrhythmika einen höheren Wirksamkeitsgrad und eine vergleichbare Sicherheit aufweist. Dies zeigt sich in der Reduktion von Vorhofflimmerrezidiven, der Reduktion der Symptomlast, der Verbesserung der Lebensqualität und der verzögerten Progression von Vorhofflimmern [2, 31].

Patienten mit paroxysmalem Vorhofflimmern sollten im Rahmen der AF-CARE-Säule R [Reduktion von Symptomen durch Rhythmus- oder Frequenzkontrolle] nach der Adressierung der [C]- und [A]-Säulen über die Möglichkeit einer Katheterablation informiert werden. Jeder Patient, für den eine Katheterablation in Frage kommt, sollte über die Notwendigkeit einer holistischen Behandlung des Vorhofflimmerns informiert werden. Zudem sollten alle Behandlungsentscheidungen gemeinsam mit dem jeweiligen Patienten getroffen werden („shared decision-making“), wobei die Wünsche und Bedürfnisse des Patienten sowie die möglichen Behandlungsoptionen im Hinblick auf die jeweiligen Risiken und Vorteile zu berücksichtigen sind. Die Überlegenheit der Katheterablation als Erstbehandlung bei paroxysmalem Vorhofflimmern wurde in randomisierten Studien nachgewiesen, die relativ junge Patienten mit einer erheblichen Anzahl von Vorhofflimmerepisoden einbezogen. Daher sollten die Ergebnisse nicht uneingeschränkt auf Patienten übertragen werden, die nur wenige Episoden von paroxysmalem Vorhofflimmern aufweisen. Bei ausgewählten Patienten kann eine Rhythmuskontrolle neben Symptomreduktion und Verbesserung der Lebensqualität die Prognose verbessern, wie z. B. bei Patienten mit Herzinsuffizienz [15, 27].

Die Rhythmuskontrolle (medikamentös und/oder interventionell) sollte innerhalb von 12 Monaten nach der Diagnosestellung bei ausgewählten Patienten mit hohem Schlaganfall- oder Thromboembolierisiko in Betracht gezogen werden, um das Risiko für kardiovaskuläre Todesfälle oder Krankenhausaufenthalte zu verringern.

Bei Patienten mit Vorhofflimmern und Herzinsuffizienz mit verminderter Ejektionsfraktion, die vermutlich auf eine durch Tachykardie ausgelöste Kardiomyopathie zurückzuführen ist, wird die Katheterablation empfohlen, um die linksventrikuläre Dysfunktion rückgängig zu machen. Darüber hinaus wird bei ausgewählten Patienten mit Vorhofflimmern und Herzinsuffizienz mit verminderter Ejektionsfraktion eine Katheterablation angeraten, da diese die Hospitalisierung und Mortalität aufgrund der Herzinsuffizienz verringern kann [27].

## Sicherheit der Kardioversion

Die Kardioversion von Vorhofflimmern ist mit einem Risiko für Schlaganfall oder Thromboembolien verbunden. Das Risiko ist erhöht, wenn Patienten nicht ausreichend antikoaguliert sind oder mittels Bildgebung intrakardiale Thromben ausgeschlossen wurden. Dieses Risiko hängt von den Patientencharakteristika und der Dauer des Vorhofflimmerns ab. Die bisherige Episodendauer von 48 h, die eine frühe elektrische Kardioversion ohne Antikoagulation oder Echokardiographie zur Thrombusdiagnostik ermöglichte, wurde durch Beobachtungsstudien in Frage gestellt. Der exakte Zeitpunkt des Beginns des Vorhofflimmerns ist oft von der Eigenanamnese des Patienten abhängig. Die zuverlässige Bestimmung der Dauer des Vorhofflimmerns stellt daher eine Herausforderung dar. Im Hinblick auf die Patientensicherheit wird in den aktuellen Leitlinien eine kürzere Dauer der Vorhofflimmerepisode von 24 h empfohlen, wenn eine frühe elektrische Kardioversion bei Patienten durchgeführt werden soll, die nicht mindestens 3 Wochen lang wirksam antikoaguliert wurden oder bei denen mittels Echokardiographie kein Thrombenausschluss erfolgt ist.

Die elektrische Kardioversion ist sehr effektiv bei der Wiederherstellung des Sinusrhythmus und kann in verschiedenen klinischen Situationen von Nutzen sein. Im Notfall wird die elektrische Kardioversion bei Patienten mit instabiler Hämodynamik sofortig empfohlen. Die elektrische Kardioversion kann auch dann eingesetzt werden, wenn die Symptomkorrelation mit der Rhythmusstörung nicht klar ist, oder als diagnostisches Verfahren, wenn die Vorteile der Wiederherstellung des Sinusrhythmus unklar sind. Die diagnostische Bedeutung der elektrischen Kardioversion kann auch bei Patienten mit Vorhofflimmern und eingeschränkter Funktion des linken Ventrikels von Nutzen sein, wenn eine Tachykardiomyopathie als Differenzialdiagnose in Betracht gezogen wird.

Trotz der Effektivität der elektrischen Kardioversion ist bei Patienten mit neu aufgetretenem Vorhofflimmern eine Spontankonversion in Sinusrhythmus sehr wahrscheinlich. Eine Watch-and-see-Strategie mit zunächst Frequenzkontrolle ist einer frühen Kardioversion zur Wiederherstellung des Sinusrhythmus nach 4 Wochen nicht unterlegen [19]. Ungeachtet der gewählten Strategie und der Art der Kardioversion (elektrisch oder pharmakologisch) ist es von entscheidender Bedeutung, dass die Patienten vor der geplanten Kardioversion mindestens 3 Wochen lang eine adäquate therapeutische Antikoagulation erhalten.

## Trigger-induziertes Vorhofflimmern

Ebenfalls handlungsorientiert dargestellt wird Trigger-induziertes Vorhofflimmern. Die Arrhythmie kann durch unterschiedliche Trigger in zeitlicher Nähe zu einem auslösenden und potenziell reversiblen Ereignis auftreten. Häufigste Auslöser sind ein postoperativer Zustand (insbesondere nach Herzoperation; bis ca. 30 % der Patienten) eine Sepsis (9–20 % Auftretenswahrscheinlichkeit), Alkoholkonsum, Drogenmissbrauch und chronisch-entzündliche Erkrankungen. Auch dieses Vorhofflimmern wird nach dem AF-CARE-Prinzip behandelt mit Fokus auf die zugrundeliegende Erkrankung sowie begleitende Risikofaktoren [7]. Die langfristige orale Antikoagulation sollte abhängig vom individuellen Risiko für Schlaganfälle oder Thromboembolien erwogen werden.

## Device-detektiertes Vorhofflimmern

Device-detektiertes Vorhofflimmern ist asymptomatisches Vorhofflimmern, das durch ein kontinuierliches Monitoring durch implantierbare, elektronische Geräte identifiziert wird. Device-detektiertes Vorhofflimmern erhöht das Schlaganfallrisiko, jedoch deutlich weniger als klinisches Vorhofflimmern [10, 28]. Mit zunehmender Dauer der Episoden scheint das Risiko für thromboembolische Ereignisse zu steigen [9]. Basierend auf zwei randomisierten Studien, von denen eine frühzeitig abgebrochen wurde, kann eine Antikoagulation bei Patienten mit Device-detektiertem Vorhofflimmern erwogen werden. In der ARTESiA-Studie war unter Apixaban im Vergleich zu Acetylsalicylsäure das Risiko für Schlaganfälle oder systemische Embolien niedriger, allerdings auch das Risiko für schwere Blutungen erhöht [8]. Die NOAH-Studie wurde vorzeitig beendet aufgrund von Sicherheitsbedenken (erhöhte Blutungsrate unter Edoxaban) und fehlender Wirksamkeit [12]. Wichtig ist, dass Device-detektiertes Vorhofflimmern in 6–9 % der Fälle pro Jahr in ein klinisch manifestes Vorhofflimmern übergeht und dann nach dem AF-CARE-Prinzip behandelt werden sollte.

## Screening auf Vorhofflimmern

Obgleich Vorhofflimmern eine der weltweit häufigsten anhaltenden Herzrhythmusstörungen ist, bleibt es dennoch oft unbemerkt, bis Folgeerkrankungen auftreten. Seine Prävalenz wird aufgrund der Bevölkerungszunahme, der Alterung der Bevölkerung und der verbesserten Überlebenschancen bei anderen Herzerkrankungen weiter ansteigen. Die Leitlinien enthalten Strategien für das Screening, die Früherkennung und die Primärprävention von Vorhofflimmern in der Population.

Die systematische Suche nach Vorhofflimmern in der Bevölkerung im Rahmen von Screeningprogrammen wird explizit von der Detektion während Routineuntersuchungen im Rahmen der medizinischen Versorgung unterschieden. Bei allen Personen ab 65 Jahren wird eine routinemäßige Untersuchung der Herzrhythmusstörungen während des Kontakts mit medizinischem Personal empfohlen, um Vorhofflimmern frühzeitig zu erkennen. Bei Individuen ab 75 Jahren oder ab 65 Jahren mit weiteren Risikofaktoren nach dem CHA_2_DS_2_-VA-Score sollte ein systematisches Screening mit Hilfe eines nicht-invasiven EKG-Verfahrens über einen längeren Zeitraum erwogen werden, um Vorhofflimmern rechtzeitig zu erkennen. Die endgültige Bewertung der optimalen Zielgruppen für ein Screening, der optimalen Screeningdauer, der Nutzen neuer diagnostischer und Verbrauchertechnologien (u. a. Wearables) sowie der Kosteneffektivität von Screening in der klinischen Routine oder systematischen Screeningprogrammen in der Bevölkerung ist aufgrund der noch zu geringen Datenlage nicht möglich.

## Fazit für die Praxis

Die 2024 veröffentlichten Leitlinien der ESC zum Management von Vorhofflimmern geben aktuelle, evidenzbasierte Empfehlungen für eine umfassende Behandlung von Vorhofflimmerpatienten. Diese wird durch das AF-CARE-Konzept vorgegeben, das Komorbiditäten und Risikofaktoren in den Vordergrund stellt. Es soll Symptomlast, Rezidivhäufigkeit, Lebensqualität und Prognose verbessern. Eine Antikoagulation, vorzugsweise mit einem DOAK, wird bei Patienten mit erhöhtem Risiko für Schlaganfälle empfohlen. Zur Risikoabschätzung kann der CHA_2_DS_2_-VA-Score, der auf die Geschlechtskomponente verzichtet, genutzt werden. Vorhofohrverschluss und Exklusion wird in laufenden Studien weiter untersucht und wird bei Herzoperationen bei Patienten mit Vorhofflimmern empfohlen und sollte bei endoskopischen oder Hybrid-Ablationen als Ergänzung zur oralen Antikoagulation erwogen werden. Trigger-induziertes Vorhofflimmern wird entsprechend des AF-CARE-Konzepts adressiert, genauso wie es neue Empfehlungen zum Device-detektierten Vorhofflimmern gibt. Die Indikation zur Katheterablation von Vorhofflimmern wurde gestärkt. Empfehlungen zur sicheren Kardioversion werden formuliert. Eine Patientenversion der Leitlinien wurde ergänzend erstellt und unterstützt den integrierten Behandlungsansatz.

## Data Availability

Das Manuskript beinhaltet keine Originalmaterialien oder -daten.

## Abkürzungen

CHA_2_DS_2_-VA-Score: Herzinsuffizienz, Hypertonie, Alter ≥ 75 Jahre, Diabetes mellitus, Schlaganfall/transitorische ischämische Attacke oder periphere Thromboembolie, vaskuläre Erkrankung, Alter zwischen 65 und 74 Jahren
DOAK: Direktes orales Antikoagulans
